# Super high-quality SEM/FIB imaging of dentine structures without collagen fiber loss through a metal staining process

**DOI:** 10.1038/s41598-022-06271-y

**Published:** 2022-02-11

**Authors:** Shiyou Xu, Michael Stranick, Deon Hines, Ke Du, Long Pan

**Affiliations:** 1 Colgate-Palmolive Technology Center, 909 River Road, Piscataway, NJ 08844 USA; 2 Department of Mechanical Engineering, Rochester Institute of Technology, Rochester, NY 14623 USA

**Keywords:** Scanning electron microscopy, Dental foundation training

## Abstract

Scanning Electron Microscope/Focused Ion Beam (SEM/FIB) system has become valuable and popular tool for the analysis of biological materials such as dentine structures. According to physiological and anatomical studies, dentine structures are a complicated system containing collagen fibers, nanocrystalline hydroxyapatite, and numerous networks of tubular pores. During a routine FIB milling process, collagen fibers and other organic structures are vaporized, which increases the number of pores on the milled surface of the dentine. This causes the final cross-section to be more porous than the pristine sample. Unfortunately, little attention has been paid to the collagen fiber loss and how to preserve them during a FIB milling process. In this work, we present a novel and simple approach to preserve the organic portions of the dentine structure through metal staining. By using this method, the porosity of the dentine structure after the FIB milling process is significantly reduced similar to the pristine sample. This indicates that the organic portion of the dentine structure is well protected by the metal staining. This approach enables the SEM/FIB system to generate super-high quality SEM images with less ion beam damage; and the SEM images can better reflect the original condition of the dentine structure. Further, serial energy-dispersive X-ray spectroscopy (EDS) mapping of the stained dentine structure is achieved without an additional metal coating; and three-dimensional (3-D) elemental mapping of an occluded dentine is achieved with a significantly reduced data acquisition time.

## Introduction

Dentine, a complicated system containing macro, micro, and nano-features, is a part of the tooth which plays a critical role in responding to hypersensitivity, the mechanical strength of the tooth, and overall oral hygiene^1–3^. Dentine is a matrix of collagen fibers, bundles of nanocrystalline hydroxyapatite, and networks of dentine tubules^4–6^. The composition of dentine is approximately 70% inorganic structures, 20% organic structures, and 10% water^7–10^. The networks of dentine tubules are radially distributed along the width of the dentine and span from the inner dental pulp to the interface between the dentine and enamel^11–13^ with smaller tubes connecting to the adjacent tubules. Upon exposure to stimuli, such as a temperature or pH change, there is a rapid displacement of the fluid inside of the dentine tubules. These structures can become exposed to the changing stimuli through erosion of the enamel layer or recession of the gum line. This triggers the deformation of the pulp nerve fibers, resulting in a painful symptomatology that is often referred to as tooth or dentine hypersensitivity^14–16^. Dentine hypersensitivity, a sharp pain resulting from chemical or temperature stimuli, has been an increasing manifestation that affects a large portion of the world’s population^17–20^. The basic structure of the dentine is of great interest because it is relevant to the treatment of dentine hypersensitivity using dentine tubular occlusion^21–23^. Accurately imaging the dentin structure with high quality is the key not only for dentists to treat their patients but also for the oral care industry to develop their products to treat dentine hypersensitivity.

When analyzing the dentine structures and dentine occlusion, it is desirable to obtain the cross-section images along the dentine tubules such that the view of the dentine structure (with/without the occluding plugs) can be obtained. Further, when transmission electron microscopy (TEM) is used to analyze the dentine structure at the atomic level, ultra-thin sections are required^24–27^. There are several methods that can be used to obtain cross-sectional images of a dentine, such as microtomy, frozen fracture, and FIB techniques^28–30^.

The FIB technique relies on a high-energy ion beam to bombard and remove part of the target material. Thus, it is inevitable that the FIB process will generate damages and artifacts during the milling process. The artifacts may be on the top surface or on the targeted sidewall; and may include amorphization, gallium implantation, and structural modification^31–37^. When analyzing dentine structures using a FIB, either for structural analysis or a thin lamellar preparation for high-resolution TEM analysis, the high energy ion beam can cause severe damages to the organic portions and etch away collagen fibers, leaving holes and/or small channels in the dentine. The holes and small channels formed by the etching of the ion beam will introduce artifacts which could add more channels or pores. These small channels will further propagate the severe curtaining effects observed during TEM sample preparation. Even though dentine structures have been widely studied by electron microscopy, to the best of our knowledge, there has been no report focusing on the damages to the organic portions of the dentine caused by the FIB, especially on collagen fiber loss, and how to stabilize the dentine structures during the milling process. Further, most of the reported SEM images did not reveal the subtle structures of the dentine, such as the pores in the intertubular regions, because the resolution was not high enough. Thus, the SEM images could not fulfill all of the requests for product development for consumers, as well as for dentists to diagnose oral care issues in their patients.

In the present study, we take advantage of a metal staining process onto the dentine structures for SEM/FIB analysis. By simply staining the dentine structure in RuO_4_ vapor, the collagen fibers were well preserved during the FIB milling process, and the number of pores generated by the damage from the ion beam was significantly reduced. As a result, super-high resolution SEM images of the dentine structures were obtained from both the top surface and cross-sections. The high-resolution SEM images reveal all the detailed information of the dentine structures, especially for the collagen fibers. Further, after staining the occluded dentine structure using RuO_4_, energy-dispersive X-ray spectroscopy (EDS) mapping was performed without any additional metal coating; and elemental maps of serial slices were obtained without coating each slice, significantly simplifying the EDS mapping acquisition process and the total acquisition time.

## Results

**Figure 1:**
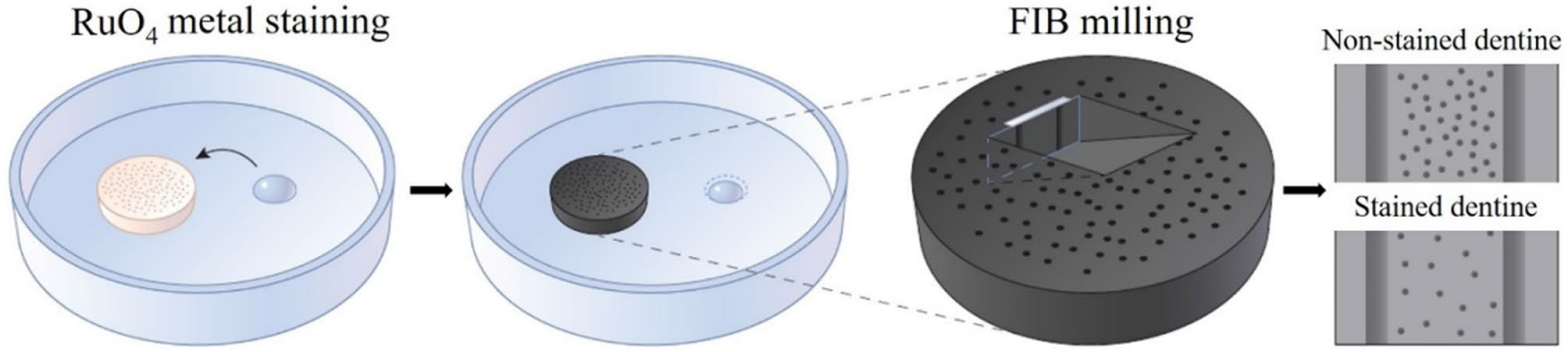
Experimental procedure for the protection of collagen fibers in FIB milling: the dentine disk was exposed to RuO_4_ vapor, followed by FIB filling. Low porosity is achieved due to the reduction of collagen fibers.

**Figure 2:**
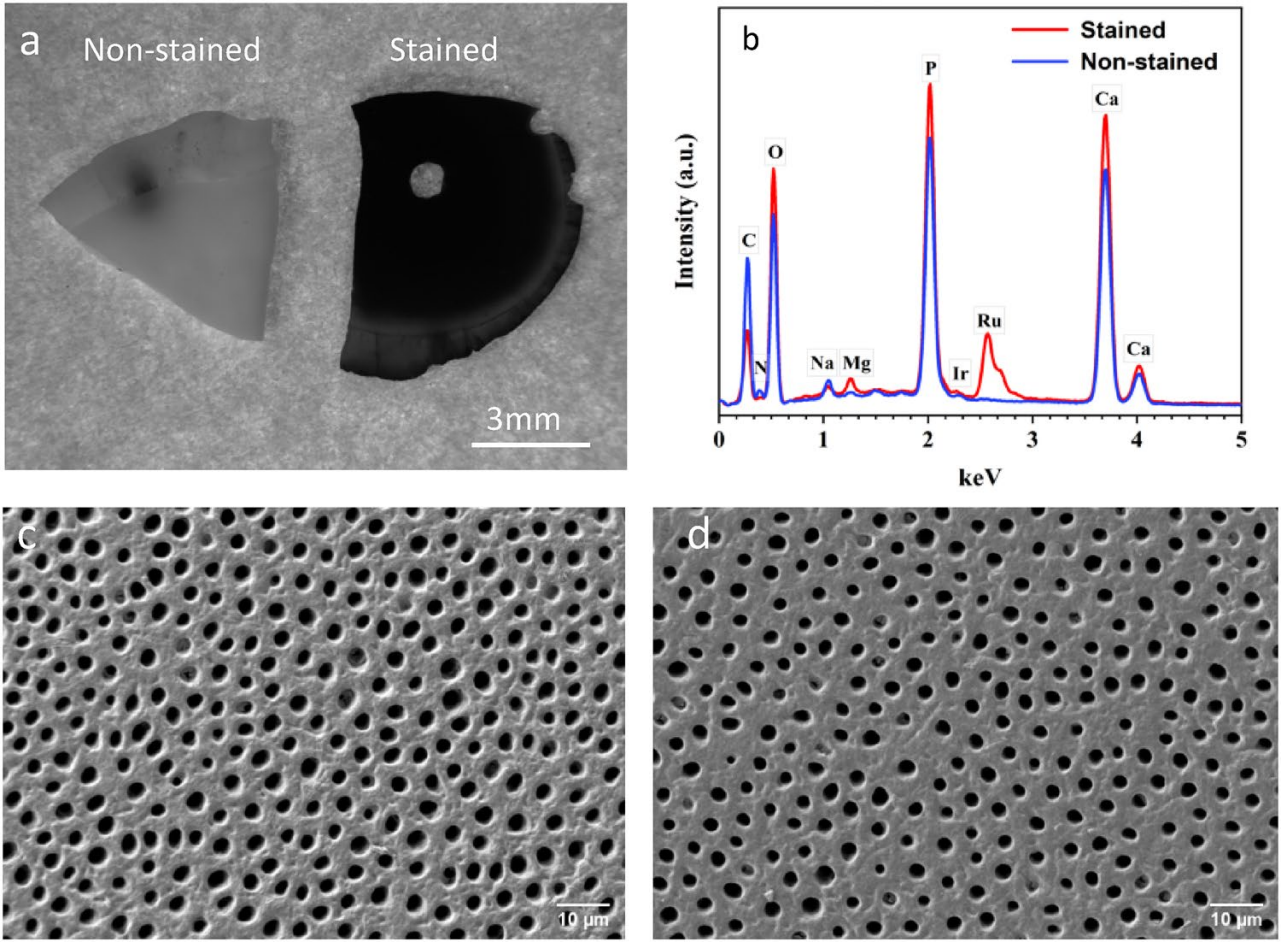
(**a**) Optical micrograph and (**b**) EDS spectrum of the non-stained and stained dentine disk. SEM image of the top surface of the (**c**) non-stained and (**d**) stained dentine disk.

Figure 1 illustrates the experimental procedure for the metal staining process and the FIB milling process. The dentine structure before and after metal staining is shown in Fig. 2a. After the RuO_4_ staining process, the color of the dentine disk turns from grey to black, indicating the uniform staining over the top surface. As shown in Fig. 2b, the non-stained dentine sample contains C, N, O, Na, Ca, Mg, P and S, while the stained dentine sample contains the same chemical species, with the addition of Ru, which indicates its presence on the surface. Under SEM imaging, the morphology of the dentine surface has no significant change before and after staining process, as shown in Fig. 2c,d, respectively.

**Figure 3:**
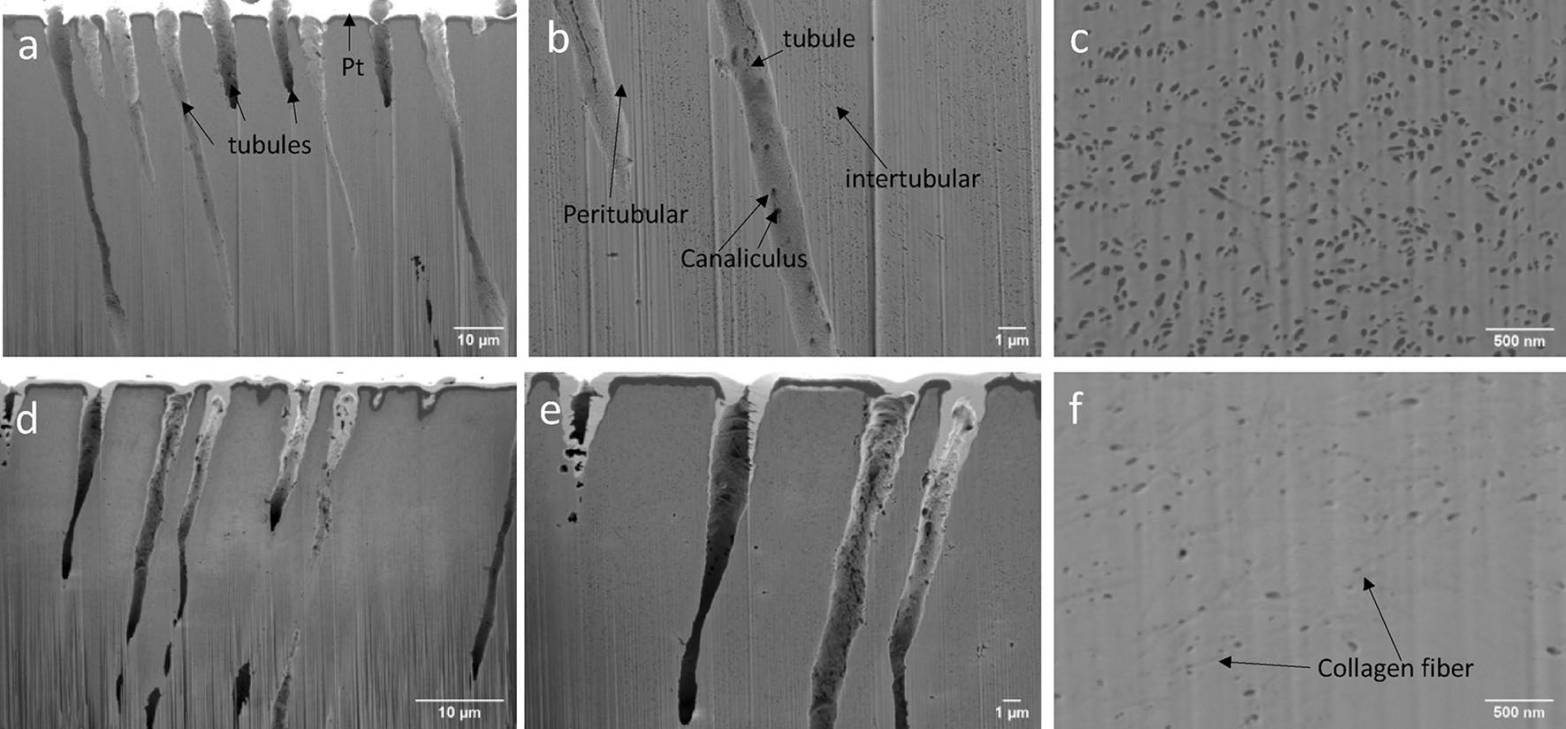
SEM images of the non-stained and stained dentine disk: (**a**), (**b**), and (**c**) are the cross sections of the non-stained dentine disk at different magnifications; (**d**), (**e**), and (**f**) are the cross-sections of the stained dentine disk at different magnifications.

After confirming that the RuO_4_ staining process does not affect the macrostructures, we then explore how the staining process affects the microstructures inside the dentine disk. Figure 3a–c are the cross-sectional views of the non-stained dentine sample at different magnifications; and Fig. 3d–f are the cross-sectional views of the stained dentine sample at different magnifications. The nanopore density of the intertubular region for the non-stained sample is significantly larger than that of the stained sample, indicating that without metal staining, the collagen fibers are destroyed and therefore show more porous structures. In addition, the preserved collagen fibers are clearly observed with a high magnification (Fig. 3f).

**Figure 4:**
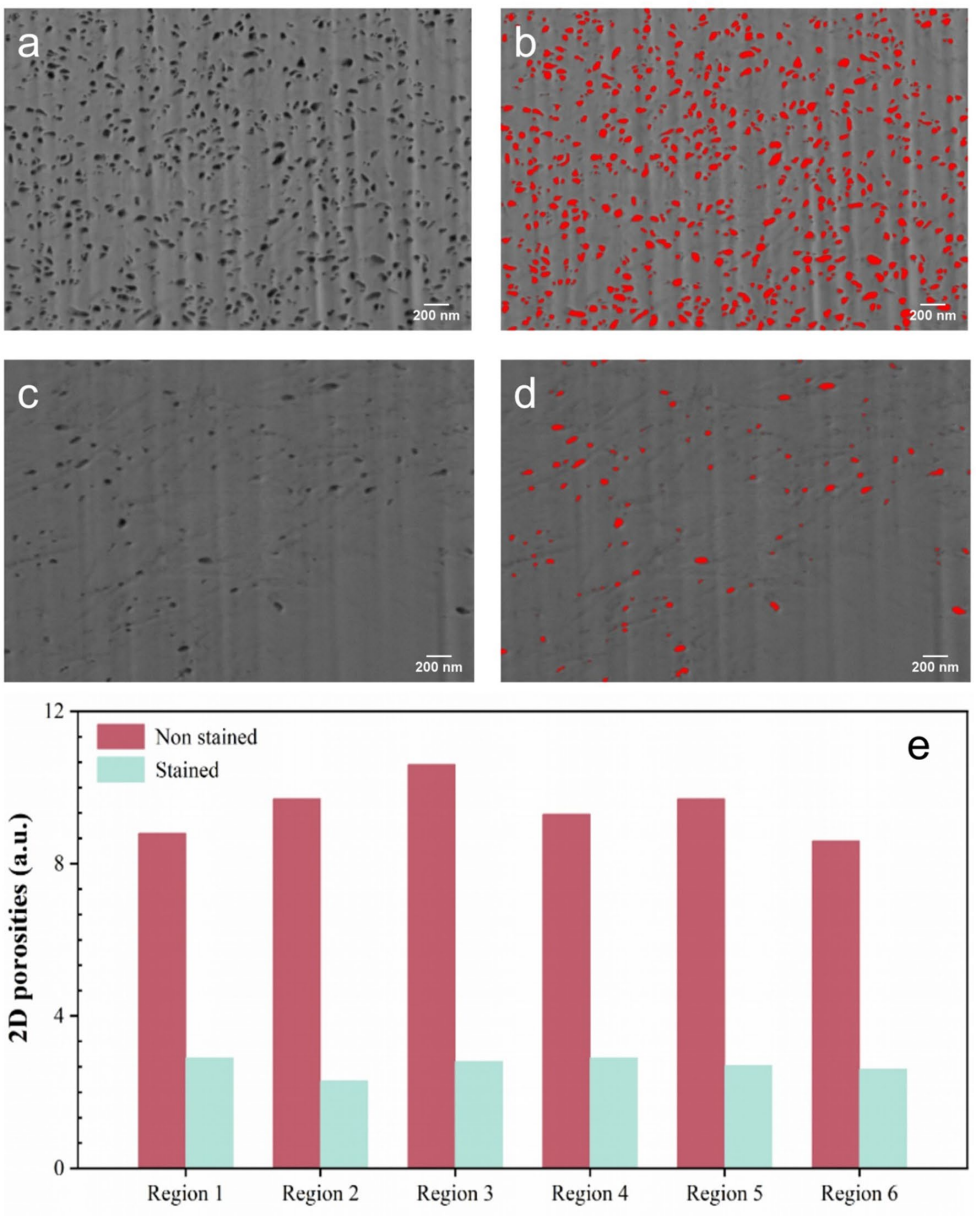
(**a**) Original and (**b**) Segmented SEM image of the intertubular region of the non-stained dentine disk; (**c**) Original and (**d**) Segmented SEM image of the intertubular region of the stained dentine disk (scale bar is 200 nm).

The porosity of the intertubular regions of the dentine was quantified by calculating the two-dimensional (2-D) surface area ratio of the pores using a commercially available program (ImageJ). The non-stained sample is shown in Fig. 4a and the pores are labeled with red dots (Fig. 4b). On the other hand, the porosity of the stained sample is much less, as shown in Fig. 4c,d. Figure 4e summarizes the porosity of 6 different intertubular regions of the non-stained and stained dentine disks. The average porosity of the dentine is reduced from 8.6–10.6 to 2.3–2.9, which is a ~ 3.5 times decrease. These results confirm that the staining process is able to preserve the collagen fibers in the dentine disks.

**Figure 5:**
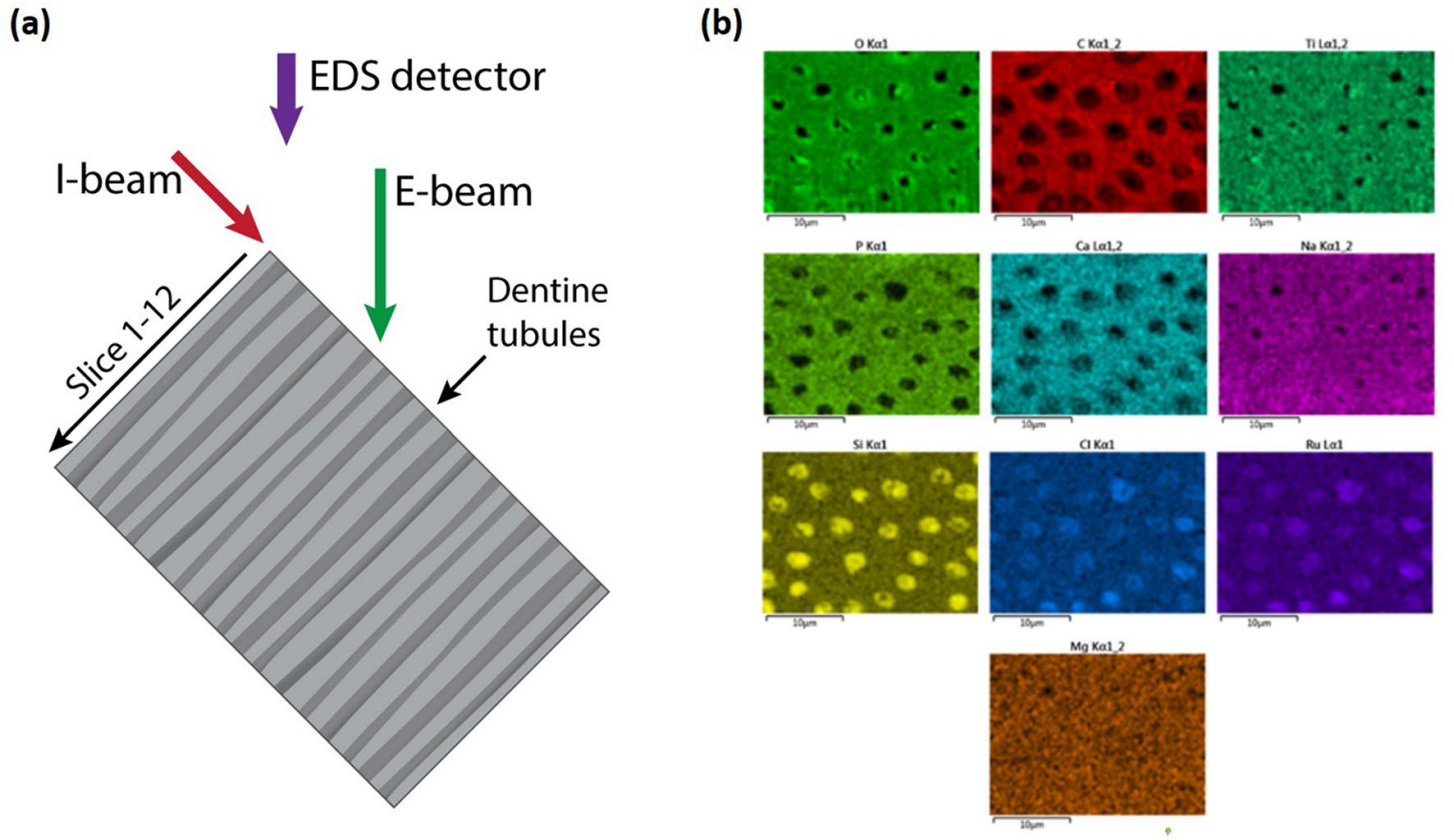
(**a**) Procedure for obtaining the elemental maps of the treated dentine at different depths. (**b**) A typical EDS mapping of the stained dentine disk at a mill depth of 2 µm, showing the presence of O, C, Ti, P, Ca, Na, Si, Cl, Ru, and Mg.

Besides the preservation effect, the metal staining process also reduces the beam sensitivity when analyzing the dentine structures. To demonstrate this, we used EDS to obtain elemental maps at different depths of the dentine tubules, and reconstructed the images as a 3-D volume of a certain element to show the occlusion efficacy of a toothpaste. Figure 5a illustrates a schematic of the setup used for acquiring elemental maps of a metal stained and toothpaste treated dentine disk. As shown in Fig. 5a, the dentine disk is vertically mounted on a SEM stub and tilted to 54º thereby the ion beam (I-beam) is parallel to the treated-side of the dentine disk and has a 36° angle with the electron beam (E-beam). The EDS detector is used to obtain the characteristic X-ray of the elements in the sample. The position of the EDS detector in Fig. 5a is for illustrative purpose only as in the experimental setup, the EDS detector overlaps the I-beam column. After acquiring an elemental map on the surface of the dentine disk, a slice with a desired thickness is milled away by I-beam (FIB). Then, another elemental map is obtained on the new surface. The milling-mapping process is repeated along the direction of the arrow until the desired total milling thickness is obtained. A typical EDS mapping of the stained dentine sample at a milling depth of ~ 2 µm is shown in Fig. 5b. This mapping indicates that all of the expected elements are present in the treated dentine. The elemental map also shows the presence of silicon in the dentine tubules, as the dentine was treated with silica-containing toothpaste (Colgate Total^@^ SF) before EDS mapping.

**Figure 6:**
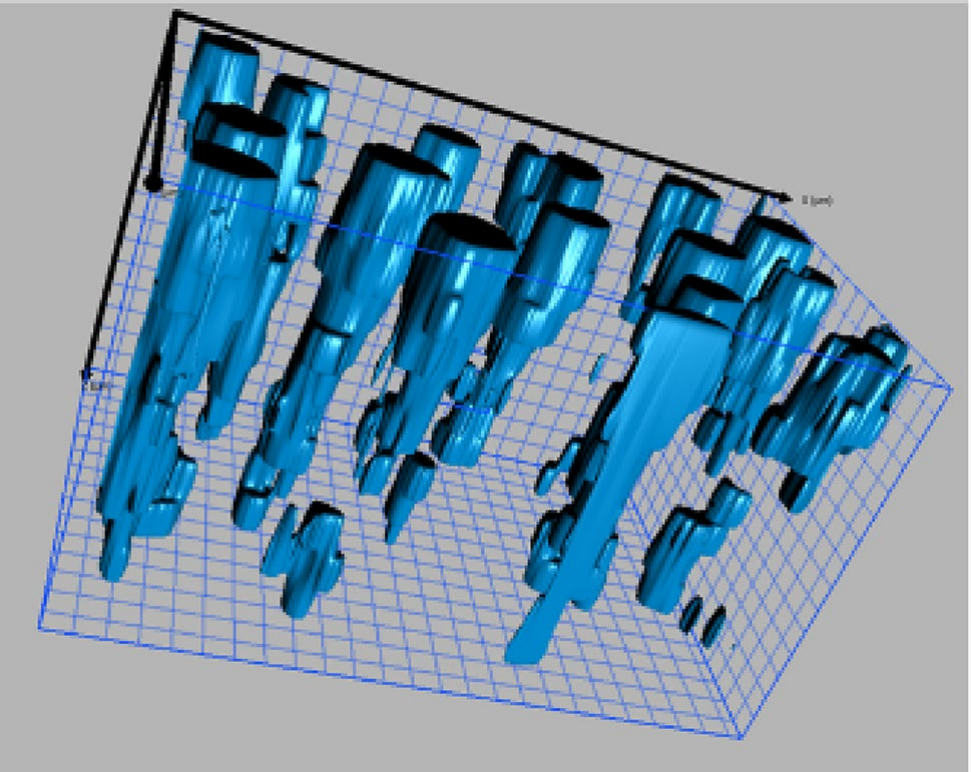
3-D EDS image of the silica occluded in the dentine tubules, grid size: 1 µm.

Figure 6 shows a 3-D image of the toothpaste treated dentine disk by reconstructing 12 silicon elemental maps at different milling depths. Referring to Fig. 5a. each elemental map is obtained by taking an elemental map after milling 1 µm deep into the dentine sample along the length direction of the dentine tubules until all 12 slices were obtained. The 3-D image of silicon inside of the dentine tubules, which corresponds to the silica from the toothpaste used to treat the dentine, can clearly show the efficacy of the toothpaste for treating dentine hypersensitivity by occluding the dentine tubules. A video of the silicon filling dentine structure is shown in the supplement information.

## Discussion

RuO_4_ is a commonly used staining agent for electron microscopy as it can fix the tissue membrane for different organs. RuO_4_ reacts strongly with proteins, glycogen, monosaccharides, olefins, sulfides, primary and secondary alcohols, and aldehydes and thus can be used to protect the organic portions of the dentine disk, especially collagen fibers. It is worth mentioning that, to the best of our knowledge, no literature has reported the usage of RuO_4_ staining on analyzing dentine tubules with such high resolution and detail, which is significant for electron microscopy-based analysis of dentine structures. The high-resolution images provide the fundamentals for research on tooth structure and the development of oral care products.

Since the intertubular regions have more collagen fibers, the porosity of the intertubular region is used to evaluate the stabilizing effect of the staining process. The SEM images of the cross-sections of the stained dentine show the preserved collagen fibers, indicating that the staining process not only preserves the collagen fibers, but also stains them to increase the contrast. Collagen fibers are the major portion of organic material within the dentine structure, which is critical for the understanding of many aspects of dentine: alteration with disease, aging, and restorative treatments such as dentine bonding. Thus, the preservation of collagen fibers during cross-sectional analysis provided real images of the dentine structure that will help us to link dentine structure properties with the study of oral infection, such as how certain dentifrices prevent bacteria attachment and biofilm formation.

TEM is a powerful tool for the analysis of the nanocrystalline structures in dentine. The key for successfully imaging the dentine structure with TEM is to prepare a thin section of the dentine using a FIB. Since collagens in the dentine comprise a relatively high ratio (up to 30%), they will be vaporized and form pores during the FIB process if not properly protected. These pores are artifacts and do not reflect the original structure of the dentine. Further, these pores will cause curtaining issues and large electron transparent areas may not be obtained. The metal staining process resolves these issues by eliminating the creation of artifacts.

Occluding dentine tubules is one of the two major methods for treating dentine hypersensitivity^17^. EDS is a readily available method for confirming the elemental information of the material occluding in the dentine tubules. However, a relatively high accelerating voltage of the SEM is required to generate characteristic X-ray signals and a high current to generate enough signals to increase the signal-to-noise ratio, which causes a charging issue on non-conductive samples, especially organic–inorganic composites such as dentine. Staining the dentine structure with RuO_4_ will increase the conductivity and reduce the beam sensitivity of the dentine sample. Thus, when taking elemental maps of the sample with an accelerating voltage of 10 kV, the charging issue is not as severe as it would be without staining; and the acceptable elemental maps can be obtained. Accordingly, a series of elemental maps can be obtained without coating conductive layers on the cross-sections during the data acquisition process. This significantly reduces the acquisition time of the EDS mapping process because the sample does not need to be taken out of the high vacuum chamber for the metal coating process between acquiring two sets of elemental maps. For example, it can save 36 h for collecting 12 sets of elemental maps from 12 slices. Therefore, staining the dentine sample may make the automatic EDS mapping acquisition at different milling depths take less time to generate a full-scale 3-D image. We show that acceptable quality of elemental maps of the cross-sections of the dentine disk treated with Colgate Total SF^@^ toothpaste can be obtained. The silicon maps at different milling depths can be reconstructed as a 3-D model to study the effectiveness of the dentine hypersensitivity treatment method.

This metal staining method can also be applied to other organic–inorganic composite structures. RuO_4_ is advantageous since it is less toxic than osmium tetroxide and the staining process can be performed through vapor staining. Also, the RuO_4_ solution does not need to be in direct contact with the sample and will not change the macroscopic structure of the sample. Thus, for TEM and/or SEM/FIB analysis, the simple RuO_4_ vapor staining process can be performed to stabilize the samples, thereby reducing the charging effect and enhancing the image quality. The high quality images will help scientists to innovate new products for oral care, and allow dentists to better understand dentine structures and provide better treatment for their patients. The results here will also be valuable in updating current textbooks for training dentists and other oral care professionals. These images will help us to make a demo, not just for education, but also fundamental and application research in academia and industry.

## Materials and methods

### Preparation of the dentine specimens

Human teeth were mounted on a dicing saw (Buehler IsoMet High Speed Pro, Buehler) and cross-sectioned with a thickness of 600 µm. The obtained slices (4 disks) were sanded and polished on a polishing grinder (EcoMet III, Buehler) with a polished cloth having a polished solution with size of 1 µm. The polished disks were sonicated in deionized water and etched with 1% citric acid to remove the smeared layer.

### Dentine occlusion treatment procedure

The surface of the dentine disks was treated with a toothpaste slurry by brushing for 30 secs with a microbrush. The toothpaste slurry was created by using one part phosphate buffer saline (PBS) solution and three parts Colgate Total^@^ SF Toothpaste. After brushing, the dentine disks were placed in 30 mL of PBS for 15 min and stirred at 130 rpm. Finally, the dentine disks were rinsed and dried. This entire procedure was repeated five times.

### Metal staining procedure

To reduce the variation between dentine disks during the comparison of before and after the staining process, the dentine disks were cut into two halves: one half was used as a control (without staining process), the other half was placed in a petri dish with the polished/treated side facing up. Drops of RuO_4_ solution (0.5%, Electron Microscopy Science) were placed adjacent to the dentine disk (not in contact with the sample), and the petri dish was covered and sealed. The staining process was performed overnight (16 h).

### Top surface analysis

After the metal staining process, the non-stained and stained dentine disks were imaged by an optical microscope and SEM (Zeiss XB540, Carl Zeiss). The elemental information for the dentine disks were obtained using EDS (Oxford, Xmax80). For SEM imaging, the samples were not coated with a metal layer. For EDS analysis, the dentine disks were coated with a 10 nm Au/Pd layer using a sputter coating system (Leica ACE 600). The accelerating voltage of the SEM was set at 1 kV, the beam current was set at 100 pA, and the dwell time was 800 ns. The pixel resolution of the SEM images for the porosity analysis was 3.5 nm by 3.5 nm. For EDS analysis, the beam voltage was set at 10 kV and the beam current was set at 2 nA.

### FIB/SEM imaging of the cross-sections

After analyzing the top surface with SEM/EDS, the dentine disks were further coated with a 90 nm Au/Pd layer. The cross-sections of the dentine disks were obtained by using a FIB and imaged by SEM. The FIB milling was performed by tilting the sample to 54 degrees, at which the ion beam bombarded perpendicular to the sample surface. The electron gun of the Zeiss XB540 system has a tandem structure (Gemini II column) with a Schottky field emission source, which enables the SEM to image the cross-sections of the samples at a low voltage without a metal coating. The accelerating voltage of the SEM for imaging the cross-section was set at 1 kV and the beam current was set at 100 pA. The FIB was operated with a liquid gallium ion source at a voltage of 30 kV. A Pt layer with a thickness of 1 µm was coated on the dentine surface to protect the sample using the FIB before the ion milling process. An ion beam current of 1.5 nA was used to cut a large window to view the cross-section and 50 pA was used to polish the cross-section.

### FIB/SEM/EDS mapping of serial slices

Twelve slices of elemental maps were collected manually. The top surface of the treated dentine was mapped first. Then, a slice with a thickness of 1 µm was milled away, and another EDS mapping process was performed. The milling-EDS mapping procedure was repeated 12 times to obtain all the 12 slices of the elemental maps. In total, 12 µm of the dentine disk was removed from the dentine surface. An ion beam with a current of 50 pA was used to mill the slice. The accelerating voltage for the EDS mapping was set at 10 kV and the beam current was set at 2 nA. The milling length of each slice was set as 40 µm, the milling depth for each slice was set as 25 µm, and the EDS mapping area was set as 28 µm by 21 µm.

### Image processing and 3D reconstruction

ImageJ^38^ was used to obtain the 2-D porosity of the cross-section. The particle analysis module was used to obtain the total area of the pores. Before performing the particle analysis process, the SEM images were smoothed for one time. ImageJ was also used to convert the color images of the EDS mapping to a black-white image for 3-D reconstruction. The 3-D reconstruction of the EDS mapping was performed using DragonFly^@^.

All methods were carried out in accordance with relevant guidelines and regulations. All experimental protocols were reviewed and approved by the Comitato Etico Romano Institutional Review Board. The samples were from male and female adults from the ages of 18 through 70 years (inclusive) and all participants signed an informed consent form.

## Supplementary Information

Supplementary Video 1.

Supplementary Information.
