# Supplementary material for: Super high-quality SEM/FIB imaging of dentine structures without collagen fiber loss through a metal staining process
Source: Sci Rep. 2022 Feb 11;12:2369. doi: 10.1038/s41598-022-06271-y (PMC8837798; doi:10.1038/s41598-022-06271-y)
Supplement: Supplementary file 2 — Supplementary Information. [file 41598_2022_6271_MOESM2_ESM.docx]

**Supplemental material**

**Super high-quality SEM/FIB imaging of dentine structures without collagen fiber loss through a metal staining process**

Shiyou Xu^1*^, Michael Stranick^1^, Deon Hines^1^, Ke Du^2^ and Long Pan^1^

*1. Colgate-Palmolive Technology Center, 909 River Road, Piscataway NJ 08844 USA*

*2. Department of Mechanical Engineering, Rochester Institute of Technology, Rochester, NY 14623, United States*

Video of the 3D visualization of dentine occlusion obtained by FIB/EDS from the metal stained dentine disk (Blue: occluded material-Si, and Gray-dentine)
